# A Hybrid Method for Pancreas Extraction from CT Image Based on Level Set Methods

**DOI:** 10.1155/2013/479516

**Published:** 2013-08-26

**Authors:** Huiyan Jiang, Hanqing Tan, Hiroshi Fujita

**Affiliations:** ^1^Software College, Northeastern University, Shenyang 110819, China; ^2^Graduate School of Medicine, Gifu University, Yanagido, Gifu 501-1193, Japan

## Abstract

This paper proposes a novel semiautomatic method to extract the pancreas from abdominal CT images. Traditional level set and region growing methods that request locating initial contour near the final boundary of object have problem of leakage to nearby tissues of pancreas region. The proposed method consists of a customized fast-marching level set method which generates an optimal initial pancreas region to solve the problem that the level set method is sensitive to the initial contour location and a modified distance regularized level set method which extracts accurate pancreas. The novelty in our method is the proper selection and combination of level set methods, furthermore an energy-decrement algorithm and an energy-tune algorithm are proposed to reduce the negative impact of bonding force caused by connected tissue whose intensity is similar with pancreas. As a result, our method overcomes the shortages of oversegmentation at weak boundary and can accurately extract pancreas from CT images. The proposed method is compared to other five state-of-the-art medical image segmentation methods based on a CT image dataset which contains abdominal images from 10 patients. The evaluated results demonstrate that our method outperforms other methods by achieving higher accuracy and making less false segmentation in pancreas extraction.

## 1. Introduction

Pancreas extraction is a highly demanded tool for computer-aided diagnosis, since it is the fundamental step for further medical image processing such as pancreatic cancer analysis, pancreas lesion detection, and pancreas three-dimensional visualization. Pancreatic disease like pancreatic cancer has high mortality rate and is difficult to timely check out and treat [1]. Patients are commonly examined using abdominal computerized tomography, and pancreas extraction from CT image can improve a variety of clinical applications. However, medical images are often impacted by noise and distortion, which causes difficulties to apply conventional segmentation methods, such as edge detection methods and region growing methods [2] to extract pancreas.

Various semiautomated methods are proposed to solve the segmentation problem. A well-known active contour model called snake [3] is proposed. This model first initializes a dynamic parametric contour around the object; then the curve evolutes to objective boundary under the impact of an energy function which contains terms of bending force, rigid force, and image force. However it is sensitive to the initial conditions and has topological problem. 

To overcome this problem, the level set approach has been proposed by Osher and Sethian [4]. Level set method embeds active contour as zero level set of a time-dependent level set function [5], and evaluation of contours is implicitly achieved through updating level set function by minimizing an energy function. Level set methods can represent complex topology of contours and handle topological changes in a natural and effective way. Inspired by these advantages, many classical level set framework-based segmentation methods are proposed by researchers. Among these models, the geodesic active contour (GAC) [6] model is represented as a paradigm for boundary-based segmentation methods, and C-V model [7] is regarded as a paradigm for region-based segmentation methods.

GAC model introduces a geodesic distance term, which contributes to pulling back the contour when it crosses the boundary of the object. This model partly reduces influence of weak boundary. C-V model is a globally optimized active contour model based on region information. It is more resisted to noise and suitable for global segmentation of weak gradient images. In addition, additive operator splitting (AOS) approach [8] is used in these models to reduce numerical errors in evaluation. Since level set methods usually need amount of computation, some well-known fast-marching methods [9–11] were proposed to speed up the processing. Moreover, a hybrid level set method [12] which combines both boundary and region information to achieve segmentation result was developed in present study. It utilizes a predefined parameter to indicate the lower bound of the gray level of the target object in region term. Its boundary term is similar with the one in GAC method. However the predefined parameter is not easy to be accurately defined and reinitialization of zero level set is needed.

Up to now, segmentation of pancreas region in CT images is still a big challenge. Surrounding of pancreas is usually complicated. Pancreas is always connected to neighbor organs and its boundary is fuzzy, even cannot be visually distinguished in most cases. The low contrast between pancreas and surrounded organs causes huge difficulties to extract the pancreas. All aforementioned level set methods have a problem of leakages into nearby tissue in segmentation results. 

This paper proposes a novel hybrid method for pancreas segmentation. The main purpose is to overcome the leakage problem, get better accuracy, and improve time efficiency. The main contribution of our method is the proper selection and combination of level set methods. A fast-marching level set method not only improves the time efficiency but also generates an optimal initial pancreas region to solve the problem that level set methods are sensitive to the initial contour location. Moreover, the modified regularization level set method ensures the accuracy of segmentation. The novelty in our method is that an energy-decrement algorithm and an energy-tune algorithm are proposed to modify level set methods. The energy-decrement algorithm optimizes fast-marching level set and reduces the leakage probability of the initial pancreas region. The energy-tune algorithm optimizes original distance regularization level set method, regards initial pancreas region as energy source to turn energy distribution of edge feature, and finally overcomes the over segmentation problem.

The accuracy of the proposed method is compared to region growing method [2], GAC model [6], C-V model [7], fast-marching method [9], and combined level set method [12]. Three measures, (1) false-positive error (FPE), (2) false-negative error (FNE), and (3) the similarity index (SI), are used as evaluated standards of accuracy. Moreover, pancreas regions which are manually extracted by experienced radiologists are taken as the golden standard.

The rest of this paper is arranged as follows. The proposed method is explained in Section 2. Dataset used in this paper is introduce in Section 3. Results and discussion of our method is presented in Section 4.  Section 5 concludes this paper and expounds our future work.

## 2. Materials and Methods

The novel hybrid pancreas segmentation method combines a first-marching level set method and a modified distance regularized level set (MDRLS) method. The MDRLS method exploits a distant regularized level set evolution (DRLSE) scheme proposed in [13]. Since MDRLS eliminates the need of reinitialization of level set function via inherently maintaining a signed distance profile near the zero level set, it is able to provide accurate numerical calculation in level set evolution. Moreover, an energy-tune function employed in MDRLS can weaken the bonding force which appears in weak boundary area. Therefore, the MDRLS can overcome the shortage of oversegmentation in weak boundary region and can keep a stable evolution even in complex texture surroundings of the pancreas. 

However, MDRLS needs amount of computation, and level set evolution is sensitive to initial position of the zero level set contour. A nonideal initial position reduces the accuracy of segmentation and increases the calculation time. In order to solve these problems, a customized fast-marching level set method based on multiple seeds is used to provide an optimal initial pancreas region for the MDRLS method and improves the efficiency of computing. The initial pancreas region is generated based on gradient features of pancreas, so it is adapted for pancreas texture structure. Moreover it covers different parts of pancreas region due to multiple seeds. Besides, an energy-decrement algorithm is applied to preserve the initial pancreas region from leaking into nearby tissues. The initial pancreas region generated by fast-marching method is optimal and meets the requirement of initial contour position of zero level set.

**Figure 1:**
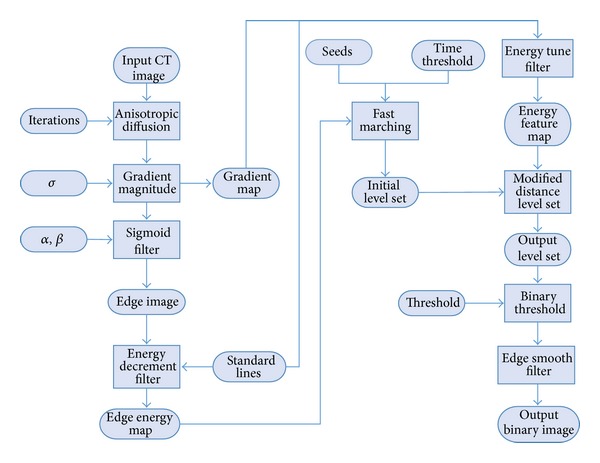
Collaboration diagram for the novel hybrid level set method applied to a segmentation task.

Therefore, the proposed hybrid level set method is time effective and able to achieve accurate segmentation results in pancreas extraction from CT images. The collaboration of the two level methods and the energy-tune algorithm is described as follows (Figure 1).

1. First an anisotropic diffusion filter is used to denoise the input CT image.
2. A gradient magnitude filter generates the gradient map of the denoised CT image.
3. A sigmoid filter forms an edge feature image based on the gradient map.
4. The energy-decrement filter optimizes the edge feature image to form an edge energy map based on standard lines.
5. Then the edge energy map is applied to modify fast-marching level set which generates an optimal initial pancreas region.
6. An energy-tune filter applies an energy-tune algorithm to adjust the gradient map of CT image to generate an energy feature map.
7. The energy feature map is used to modify distance regularized level set method. Then the actual pancreas is extracted using modified distance regularized level set method based on initial pancreas region.
8. Finally the actual pancreas is thresholded and smoothed.

The details of the aforementioned processing are described in the remaining content of this section.

### 2.1. Denoising of CT Image

 Since the intensity distribution of the pancreas is irregular due to the noise caused in the image formation stage, the abdominal CT images are necessarily denoised in preprocess. Moreover, boundaries of pancreas region which connect to neighboring organs are usually fuzzy. The important edges are easily blurred and detail of organs is significantly lost after the CT image is smoothed using a simple Gaussian filter. Therefore, a modified curvature diffusion equation (MCDE) [14] based anisotropic diffusion filter [15] is employed to reduce the influence of noise while preserving the boundaries and details of organs. 

### 2.2. Energy-Decrement and Energy-Tune Algorithm. 

**Figure 2:**
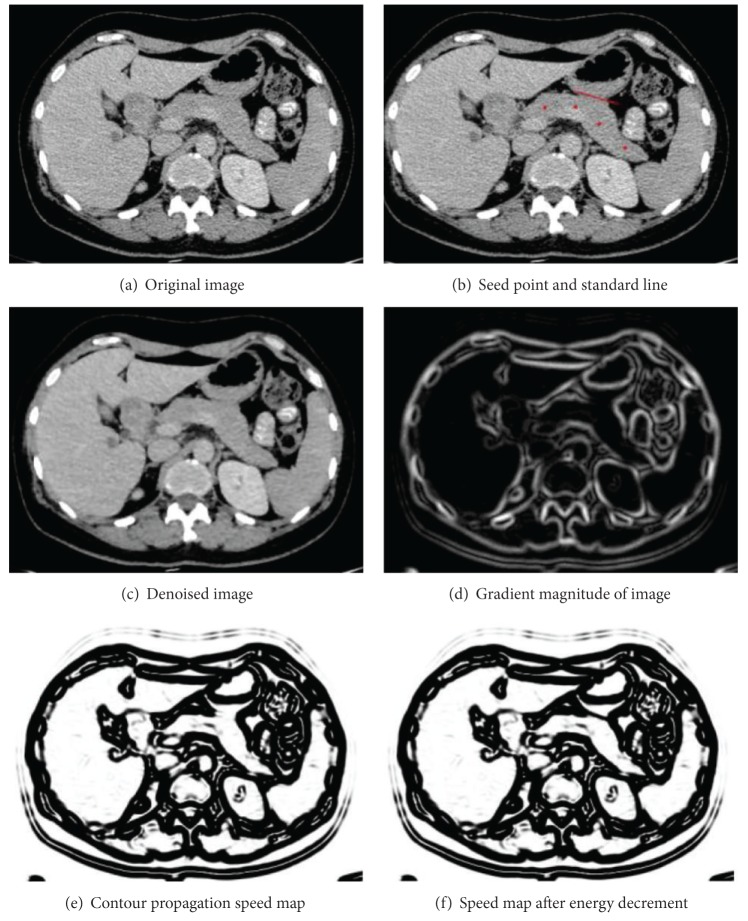
Denoising of image and feature maps of CT image. (a) Is original CT image, (b) is selection of seed points and standard line definition, (c) is denoised image processed by anisotropic diffusion filter, (d) is gradient magnitude map, (e) is edge feature map, which is employed in fast-marching level method, and (f) is energy map generated by decrement energy of speed map.

Segmentation results of fast-marching are significantly impacted by the contour propagation speed map (Figure 2(e)) and are easy to leak into nearby tissues in a weak boundary. A simple energy-decrement algorithm is proposed to prevent initial pancreas region generated by fast-marching level method beyond the authentic pancreas region. A precondition of the algorithm is that a standard line is drawn by the physician. The standard line is used to define the coupling area and separates two organs (Figure 2(b)). The energy-decrement function is defined by

(1) $$\begin{array}{c} E_{s}\left(i,j\right)=\{\begin{array}{cc} I_{\min} & D_{l}\left(i,j\right)<T_{d}\\ I\left(i,j\right) & \text{otherwise}, \end{array} \end{array}$$

where *I*_min⁡_ is the minimum value of contour propagation speed map. *D*_*l*_(*i*, *j*) is the shortest distance of pixel (*i*, *j*) to standard line. *T*_*d*_ is a distance threshold. If a distance of pixel to standard line is less than *T*_*d*_, that means it is closed to standard line, its energy is set as the minimum value of contour propagation speed map. Energy map *E*_*s*_ is used as feature map instead of contour propagation speed map in fast-marching level set method.

An energy-tune scheme is employed to support the level set function (LSF) evolution in modified distance regularized level set (MDRLS) method. An edge indicator function *g* is defined to obtain edge feature map as follows:

(2) $$\begin{array}{c} g≜\frac{1}{1+g_{m}^{2}}, \end{array}$$

where *g*_*m*_ is gradient magnitude of the CT image which has been preprocessed. This function *g* normally takes smaller value at boundaries of object than at any other location. It resists noise. Its values belong to [0,1].

Since edges located in connected regions between pancreas and its adjacent organs are weak, even fractured, the edge indicator is likely to set a large value at weak boundaries. This leads to oversegmentation in level set evolution.

Pancreas and other organs have high energy after processing by edge indicator. It is assumed that the boundary regions which have high energy are caused by energy leaking of organs. An energy-tune algorithm is proposed to decay leaked energy in boundary regions. It restrains oversegmentation caused by bonding force. It is considered that the closer a pixel is to the energy source, the more energy it obtains. The initial pancreas region generated using fast-marching is considered as energy source. Besides, the standard line is also applied to label the intensity similar area between two organs. 

The energy of each pixel that comes from energy source is defined as

(3) $$\begin{array}{c} E\left(i,j\right)=\sum_{\left(i_{0},j_{0}\right)\in R_{0}}e^{-(D(p_{i,j},p_{i_{0},j_{0}})/\sigma )}g\left(i_{0},j_{0}\right), \end{array}$$

where *E*(*i*, *j*) is energy of pixel (*i*, *j*). *R*_0_ is energy source. *D*(*p*_*i*,*j*_, *p*_*i*_0_,*j*_0__) is Euclidean distance between pixel (*i*_0_, *j*_0_) of energy source and pixel (*i*, *j*). *g* is edge feature map. The closer a pixel is to energy source, the larger energy it absorbs from the energy source. 

The energy-tune function is defined by

(4) $$\begin{array}{c} E_{t}\left(i,j\right)=\{\begin{array}{cc} \min\left(1,\frac{1}{n}\alpha E\left(i,j\right)+g\left(i,j\right)\right) & \text{if}\;\left(i,j\right)\in R_{0}\\ \max\left(0,g\left(i,j\right)-\frac{\beta E\left(i,j\right)}{D_{l}\left(i,j\right)}\right) & \text{if}\;D_{l}\leq D_{r}\\ g\left(i,j\right) & \text{otherwise}, \end{array} \end{array}$$

where *E*_*t*_(*i*, *j*) is adjusted energy of pixel (*i*, *j*). *R*_0_ is energy source. *n* is total number of pixels in energy source. *D*_*l*_ is shortest distance of pixel (*i*, *j*) to standard line. Similarly *D*_*r*_ is shortest distance of pixel (*i*, *j*) to energy source. *g* is edge feature map. *α* and *β* are parameters to control energy tune. 

The energy-tune function decays the energy of pixels closed to standard line but far from energy source, but enhances the energy of pixels that belong to the initial pancreas region. Moreover, we also propose an automatic energy-tune algorithm, which does not depend on standard line. The initial pancreas region is still regarded as energy source. A distance threshold *D*_*t*_ is defined to partition the energy tune area. If the shortest distance of a pixel to energy source is farther than threshold, its energy will be significantly decayed. Energy of pixels whose shortest distance to energy source belongs to [0, *D*_*t*_] is adjusted by the following equation:

(5) Et(i,j)={min⁡⁡(1,g(i,j)    +α1nE(i,j))(i,j)∈R0 max⁡⁡(0,g(i,j)    −1Dr(i,j)−Dt    ×βE(i,j))Dr(i,j)>Dt11+Dr(i,j)/Dtg(i,j)0<Dr(i,j)<Dtg(i,j)otherwise,

where *D*_*t*_ is the distance threshold.  *R*_0_ is energy source. *D*_*r*_ is shortest distance of pixel (*i*, *j*) to energy source. *α* and *β* are parameters to control energy tune. *g* is edge feature map.

It is specially stated that if a standard line is not drawn by the physician, the energy-decrement function cannot work. Moreover, the automatic energy-tune scheme is employed in MDRLS when the standard line is not defined by physician.

### 2.3. Initial Pancreas Region Extraction

 An optimal initial pancreas region in the denoised CT image is generated using a fast-marching level set method based on multiple seed points. The fast-marching level set method consists of five steps:

1. calculation of intensity gradient magnitude,
2. calculation of contour propagation speed map based on gradient magnitude,
3. calculation of energy map using energy-decrement algorithm based on contour propagation map,
4. calculation of time-crossing map which indicates, for each pixel, how much time it would take for the front to arrive at the pixel location,
5. generation of the optimal initial pancreas region based on time-crossing map.

First, magnitude of the image gradient at each pixel location is computed. The image is smoothed by convolving it with a Gaussian kernel and then applied a differential operator to generate gradient magnitude. An infinite impulse response filter [16] that approximates a convolution with the derivative of the Gaussian kernel is employed in the computational process.

Second, a sigmoid filter [17] is applied to calculate the active contour propagation speed map based on the gradient magnitude. Sigmoid intensity transformation is represented by the following equation:

(6) $$\begin{array}{c} I\left(x,y\right)=\left(\mathrm{Max}-\mathrm{Min}\right)\frac{1}{1+e^{-(\left(g_{m}(x,y)-\beta \right)/\alpha )}}+\mathrm{Min}, \end{array}$$

where Min and Max are the minimum and maximum values of the output value of sigmoid filter. *g*_*m*_(*x*, *y*) is gradient magnitude at pixel (*x*, *y*). *α* defines the width of the gradient magnitude range, and *β* defines the gradient magnitude around which the range is centered; they are used to control exaggerating of intensity differences between pancreas and other organs. Min is always set to 0 and Max is set to 1. 

**Figure 3:**
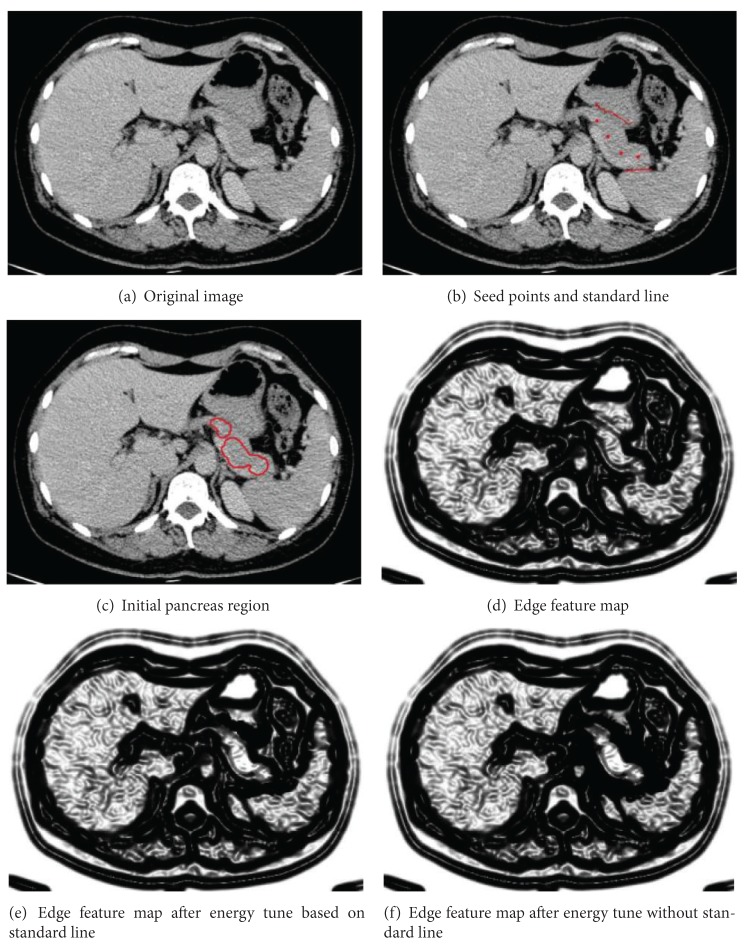
Energy-tune results based on initial pancreas region. (a) Is original image. (b) Is selection of seed points and standard line. (c) Shows initial pancreas region which is regarded as energy source. (d) Is edge feature map. (e) The energy tune based on standard line reduces the energy of pixels near standard line and enhances energy of pixels inside initial pancreas region. (f) Automatic energy tune reduces energy of pixels of which distance to energy source is larger than the distance threshold.

Third, the contour propagation map is processing using energy-decrement algorithm to form energy map (Figure 3(f)). 

Then time-crossing map which indicates the arrival time of the active contour propagation at each pixel was calculated using a fast scheme. Let *T*(*x*, *y*) be the time at which the curve crosses the point (*x*, *y*). The surface *T*(*x*, *y*) satisfies the following equation:

(7) $$\begin{array}{c} \left|\nabla T\right|E_{t}=1, \end{array}$$

where *E*_*t*_ is energy map. If standard line is not defined, *E*_*t*_ is replaced by contour propagation speed map *I*.

Finally, an optimal initial pancreas region is extracted by defining a time threshold to take a snapshot of the contour at a particular time during its evolution from the time-crossing map. 

### 2.4. Modified Distance Regularized Level Set Method

 The actual pancreas region is extracted using a modified distance regularized level set method based on the initial pancreas region. 

An original distance regularization level set evolution is proposed in [13] and the energy function of level set is defined by

(8) $$\begin{array}{c} E\left(\phi \right)=\mu R_{p}\left(\phi \right)+\beta \eta \left(\phi \right), \end{array}$$

where *μ* > 0 is a constant. *R*_*p*_(*ϕ*) is level set distance regularization term, and *η*(*ϕ*) is external force term. *R*_*p*_(*ϕ*) is defined by

(9) $$\begin{array}{c} R_{p}\left(\phi \right)≜\int_{\Omega }^{}p\left(\left|\nabla \phi \right|\right)dx, \end{array}$$

where *p* is a double-well potential function for the distance regularization term *R*_*p*_ and is constructed as

(10) $$\begin{array}{c} p\left(s\right)=\{\begin{array}{cc} \frac{1}{{\left(2\pi \right)}^{2}}\left(1-\cos\left(2\pi s\right)\right), & \text{if}\;s\leq 1\\ \frac{1}{2}{\left(s-1\right)}^{2}, & \text{if}\;s>1. \end{array} \end{array}$$

In order to decrease leakages to nearby tissues in segmentation results, a modified distance regularization level set method, based on the energy-tune algorithm, is proposed. The energy-tune algorithm is used to modify the original distance regularization level set method. The energy function *E*(*ϕ*) for the modified level set function *ϕ* : *Ω* → *ℜ* is defined by

(11) E(ϕ)=μRp(ϕ) +λ∫ΩEtδε(ϕ)|∇ϕ|dx+α∫ΩEtHε(−ϕ)dx,

where *μ* > 0 is a constant. The second energy term represents edge force which pushes the curve towards the boundaries of the object. It makes the initial contour move faster and closer to the boundaries of an object. It is minimized when the contour of zero level set is located at boundaries of object. *E*_*t*_ is the energy feature map that is used to optimize the level set function.

Moreover, in function (11) *λ* > 0 and *α* ∈ *ℜ* are coefficients to control the weight of external energy. *δ*_*ε*_ and *H*_*ε*_ are smooth functions in level set methods proposed in [18, 19]. Moreover, *H*_*ε*_′ = *δ*_*ε*_ and *ε* is set to 1.5.

(12) $$\begin{array}{c} \delta_{\varepsilon }\left(x\right)=\{\begin{array}{cc} \frac{1}{2\varepsilon }\left[1+\cos\left(\frac{\pi x}{\varepsilon }\right)\right] & \left|x\right|\leq \varepsilon \\ 0 & \left|x\right|>\varepsilon , \end{array}\\ H_{\varepsilon }\left(x\right)=\{\begin{array}{cc} \frac{1}{2}\left(1+\frac{x}{\varepsilon }+\frac{1}{\pi }\sin\left(\frac{\pi x}{\varepsilon }\right)\right) & \left|x\right|\leq \varepsilon \\ 1 & x>\varepsilon \\ 0 & x<-\varepsilon . \end{array} \end{array}$$

The third energy term represents area force which is necessarily employed to speed up the propagation motion of zero level set when the initial contour is far away from the desired object boundaries. The propagation speed of the zero level set contour would slow down when it closes to object boundaries, since energy map *E*_*t*_ takes small value at the boundaries. 

The initial pancreas region is used to construct initial level set function (LSF) *ϕ*_0_ as a binary step function. 

Consider

(13) $$\begin{array}{c} \phi_{0}\left(x\right)=\{\begin{array}{cc} -c, & \text{if}\;x\in R_{0}\\ c, & \text{otherwise}, \end{array} \end{array}$$

where *c* > 0 is a constant and *R*_0_ is the initial pancreas region. *c* is always positive in pancreas segmentation.

The level set evolution equation in MDRLS formulation is finally defined by

(14) ∂ϕ∂t=μdiv⁡(dp(|∇ϕ|∇ϕ)+λδε(ϕ)div⁡(Et∇ϕ|∇ϕ|)+αEtδε(ϕ),

where div⁡ (·) is the divergence operator and *d*_*p*_ is a function defined in [13]:

(15) $$\begin{array}{c} d_{p}\left(s\right)≜\frac{p^{\prime }\left(s\right)}{s}. \end{array}$$

The distance regularization term is able to intrinsically maintain a signed distance profile near the zero level set and eliminates the need for reinitialization of level set function. Therefore, induced numerical errors caused by reinitialization are avoided. Besides, edge-based active contour model is an advantage in optimal segmentation of local object. Thus, the edge-based active contour model in MDRLS formulation is more suitable for pancreas segmentation under the complicated surrounding due to stable and accurate numerical computation.

### 2.5. Actual Pancreas Region Extraction

 In practical pancreas region extraction process, a two-phase-segmentation scheme is employed based on the edge-based MDRLS level set method. The first phase can be seen as a high speed level set evolution, and the second phase can be seen as a high accurate zero level set contour evolution. Two iteration numbers, an interiteration number and an outeriteration number, are, respectively, applied in different phases. 

In the first phase, the zero level set is initialized as a binary step function in accordance with the function (3). Since intensity distribution of pancreas region is irregular and boundary is usually not well defined, a small coefficient *α* is set to −1 for the energy term ∫_*Ω*_ *E*_*t*_ *H*_*ε*_(−*ϕ*)*dx* in order to prevent contour from expanding too rapidly and preserve the zero level set contour from crossing the boundary of pancreas region. *λ* is usually set larger than *μ*. A relatively large weight is assigned to energy term ∫_*Ω*_ *E*_*t*_ *δ*_*ε*_(*ϕ*) | ∇*ϕ* | *dx* that means a stronger constraint force of boundary pushes zero level set curve towards boundary while limiting the oversegmentation of pancreas region. The interiteration is used to define the level set evolution time in first phase.

After first phase evolution, the zero level set contour is closed to the object boundary. In the second phase, the main purpose is to accurately extract the pancreas region. The level set evolution equation is reset as

(16) $$\begin{array}{c} \frac{\partial \phi }{\partial t}=\mu \mathrm{div}(d_{p}\left(\left|\nabla \phi \right|\nabla \phi \right)+\lambda \delta_{\varepsilon }\left(\phi \right)\mathrm{div}\left(E_{t}\frac{\nabla \phi }{\left|\nabla \phi \right|}\right). \end{array}$$

The energy term ∫_*Ω*_ *E*_*t*_ *H*_*ε*_(−*ϕ*)*dx* which is used to speed up the motion of zero level set contour is abolished by setting *α* = 0, since a high speed expanding is likely to make the contour across the object boundary and then causes oversegmentation. Level set evolution is dominated by edge force in second phase. The outeriteration is used to define evolution time.

The actual pancreas region is finally optimized by using open operation and closing operation to smooth the boundary of pancreas while keeping the original shape. The small holes inside the pancreas region are filled, and the tiny noise is eliminated. 

## 3. Pancreas Dataset 

The pancreas datasets that contain 960 CT images of pancreas that come from 10 patients are used in evolution. All images are provided by PLA General Hospital, Shenyang, China. These CT images in pancreas datasets have a resolution of 515 × 512 pixels with a thickness varied between 0.6 mm and 0.7 mm. Each image in the datasets is provided corresponding golden standard manually delineated by experienced radiologists.

## 4. Results and Discussion 

The proposed hybrid level set method was compared to confident connected region growing method (CCRG) [2], geodesic active contour method (GAC) [6], geodesic active without edge method (C-V) [7], fast-marching method (F-M) [9], and a combined edge-region level set method (CER) [12]. Our method which depends on standard line is referred to as FMDSL-SL. It is referred to as FMDSL-WSL when it is not based on standard line. Our method, GAC method, fast-marching method, and confident connected region growing method are implemented using C/C++ language. C-V method and CER method are implemented in MATLAB code. All methods run on a desktop PC with 8 GB RAM and 2.4 GHz Intel Core i7 processer. The same preprocess are applied to all methods.

### 4.1. Performance Measure Standard

 For evaluation of efficiency and accuracy, three measures, (1) false-positive error (FPE), (2) false-negative-error (FNE), and (3) the similarity index (SI), are used to measure the performance of methods. 

False-positive error [20] is defined as the ratio of the total number of extracted pancreas region pixels outside the golden standard region to the total number of golden standard of pancreas region as follows:

(17) $$\begin{array}{c} \text{FPE}=\frac{N\left(OB\right)}{N\left(G\right)}\times 100\%, \end{array}$$

where *O* represents the pixels of extracted pancreas region. *G* represents the golden standard of pancreas. *B* represents the remaining areas except the region of golden standard in the CT image. *N*(*OB*) represents the total number of extracted pancreas region pixels outside the golden standard region. *N*(*G*) represents the total number of golden standard of pancreas region. 

False-negative error [20] is defined as the ratio of the total number of golden standard of pancreas outside the extracted pancreas region to the total number of pixels of golden standard of pancreas region as follows:

(18) $$\begin{array}{c} \text{FNE}=\frac{N\left(G\right)-N\left(OG\right)}{N\left(G\right)}\times 100\%, \end{array}$$

where *N*(*OG*) is total number of pixels in intersection of extracted pancreas region and golden standard of pancreas. *N*(*G*) − *N*(*OG*) is the total number of golden standard of pancreas outside the extracted pancreas region.

Similarity index [21] is defined as the percentage of pixels in intersection of extracted pancreas region and golden standard of pancreas as follows:

(19) $$\begin{array}{c} \text{SI}=\frac{2N\left(OG\right)}{N\left(O\right)+N\left(G\right)}\times 100\%, \end{array}$$

where *N*(*O*) is the total number of extracted pancreas region. 

### 4.2. Evaluation and Comparison

 All of the state-of-the-art medical image segmentation methods and the proposed method are applied to extract pancreas region from the CT images in the same pancreas dataset. Average false-positive error, false-negative error, and similarity index are, respectively, computed for each compared method based on all segmentation results of all slices from all patients. First we calculate false-positive error, false-negative error, and similarity index for each segmentation results of all methods. Then average values of the three measure standard (Figures 6, 7, and 8) of each method are calculated based on their respective segmentation results. 

Through amount of experiment, we empirically define some values of parameters of great significance to optimize the segmentation result. In (6), *α* is set to −0.5 and *β* is set to 3. The constant *c* in (3) is set to 2. The interiteration is set between 20 and 60 and outeriteration is set to between 10 and 20 in the actual pancreas segmentation processing. Moreover, in (10) *u* = 0.2, *λ* = 5, and *α* = −1 are employed in the first phase and *u* = 0.2, *λ* = 5, and *α* = 0 are employed in the second phase. 

In this configuration of parameters, the average similarity index of all segmentation results can get a high rate (SI = 0.883). The segmentation results of different shape and acreage of pancreas are controlled by adjusting the iteration time. Moreover, the parameters can be fine tuned to adapt with different CT images to get an optimal result.

**Figure 4:**
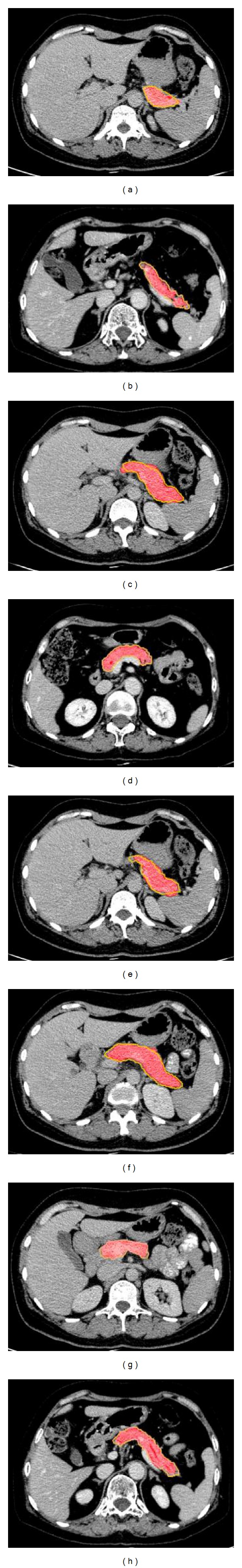
Exemplary segmentation results of our proposed hybrid method. Red regions are segmentation results using our hybrid method, and the yellow outline marks the golden standard.

Figure 4 shows some examples of segmentation results of our method. Extracted pancreas regions are complete and the edges are smooth. 

**Figure 5:**
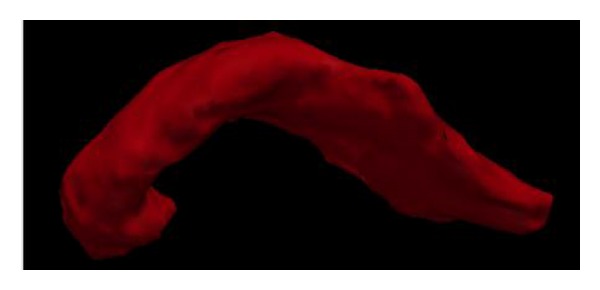
3D view of the extracted pancreas region based on the proposed hybrid level set method.

Figure 5 shows 3D view of the extracted pancreas using our proposed hybrid level set method.

**Figure 6:**
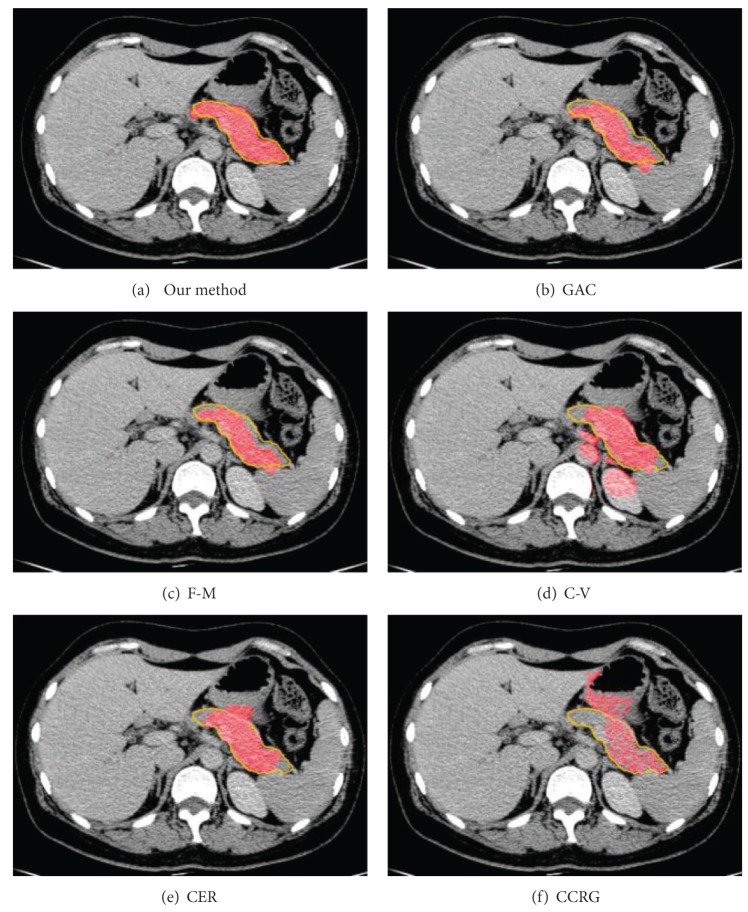
The samples of pancreas extraction results based on different methods. (a) is our method. (b) is geodesic active contour method. (c) is fast-marching method. (d) is geodesic active without edge method. (e) is combined edge-region level set method. (f) is region growing method.

Figure 6 shows examples of segmentation results of different methods to the same CT image. The red regions represent the segmentation results, and the yellow contours represent the golden standard of pancreas region.

**Figure 7:**
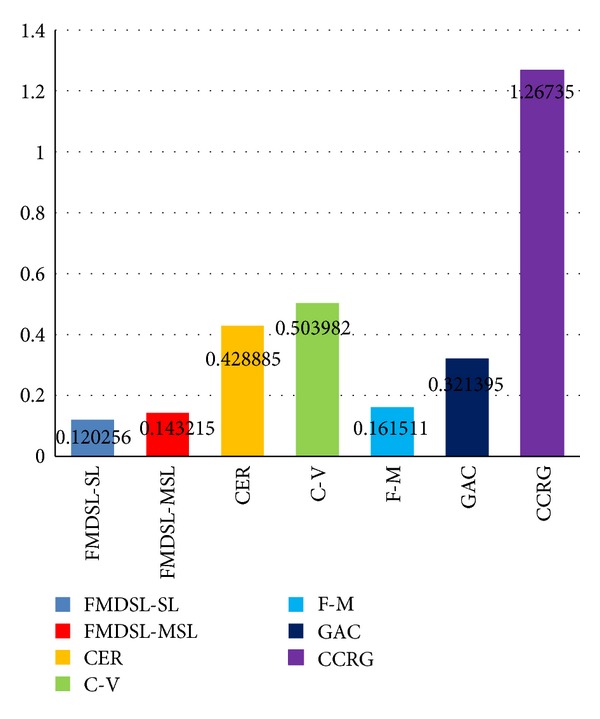
False-positive error evaluation results of our method (FMDSL-SL and FMESL-MSL), combined edge-region level set method (CER), C-V method (C-V), fast-marching method (F-M), geodesic active contour method (GAC), and confident connected region growing (CCRG) method.

**Figure 8:**
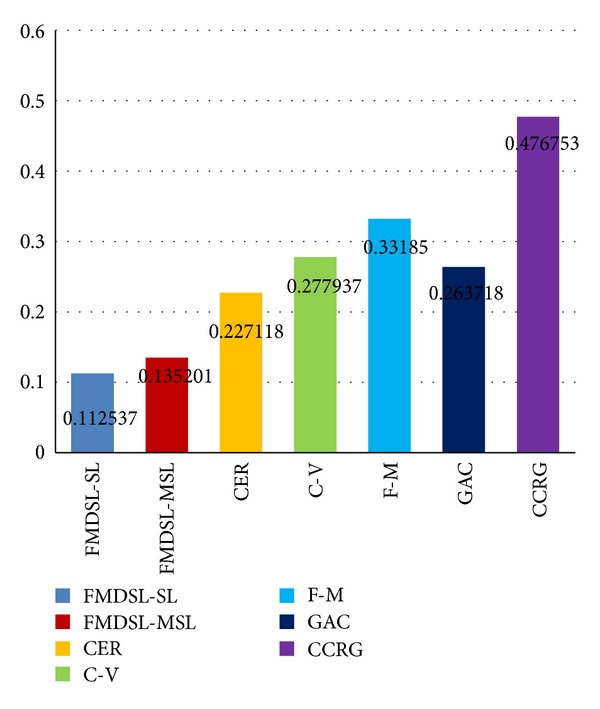
False-negative error evaluation results of our method (FMDSL-SL and FMESL-MSL), combined edge-region level set method (CER), C-V method (C-V), fast-marching method (F-M), geodesic active contour method (GAC), and confident connected region growing (CCRG) method.

**Figure 9:**
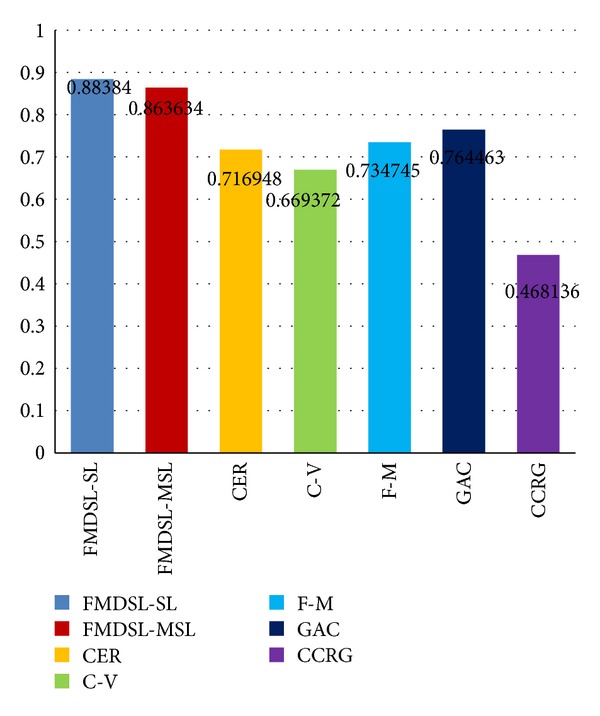
Similarity index evaluation results of our method (FMDSL-SL and FMESL-MSL), combined edge-region level set method (CER), C-V method (C-V), fast-marching method (F-M), geodesic active contour method (GAC), and confident connected region growing (CCRG) method.

Figures 7, 8, and 9 show histogram of average value of each measure standard for all compared method.  Table 1 contains accurate value of measure standards of all the compared methods. A lower false-positive error value means less pixels of background are segmented as pancreas region, and a lower false-negative error value means less golden standard of pancreas has not been extracted. Moreover, a higher similarity index means the segmentation results are more accurate. In summary, false-positive error and false-negative error are lower, and the segmentation result is more accurate. Oppositely, similarity index is higher, the segmentation result is more accurate. 

**Figure 10:**
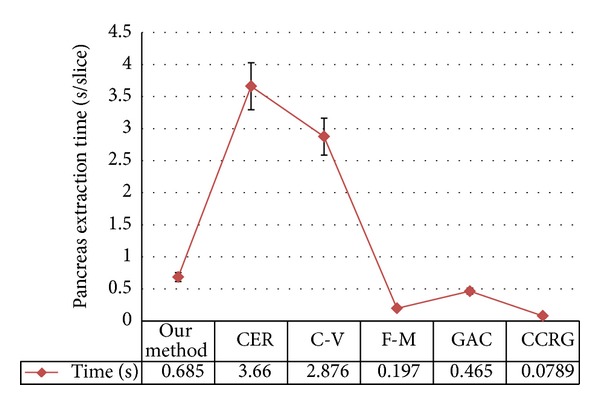
Quantitative measure of time efficiency for each method. Combined edge-region level set method refers to CER, C-V method refers to C-V, fast-marching method refers to F-M, geodesic active contour method refers to GAC, and confident connected region growing refers to CCRG method.

Figure 10 shows time efficiency of each evaluated method.

### 4.3. Discussion

 Evaluated results indicate that level set methods outperform confident connected region growing (CCRG) method in pancreas extraction.  Figure 6(f) shows that CCRG causes serious oversegmentation and recall of pancreas is not complete enough. Its average FPE = 1.26735 and FNE = 0.476 are too high and SI = 0.46 is lowest. Since in confident connected region growing method, the mean and standard deviation of intensity values are computed for all pixels which are included in the region and then they are used to define a range around the mean. Neighbor pixels whose intensity values fall inside the range are accepted and included in the region. This rule makes the intensity similar area around pancreas easy to be classified as pancreas region. This causes serious oversegmentation which is difficult to control. 

All evaluated level set methods can be classified into three types: (1) edge-based level set methods include our method, fast marching method, and geodesic active contour method, (2) region-based level set method: C-V method, and (3) the level set method combined edge and region information (CER). The evaluated results show that edge-based level set methods are more effective for single organ segmentation from a medical image which contains many other organs. Since the C-V method (FPE = 0.5, FNE = 0.278) abandons edge constraints and only pursues to achieve global optimal segmentation result, it is difficult to get an accurate result when the purpose of segmentation is to extract a local organ like pancreas. The CER (FPE = 0.43, FNE = 0.23) method utilizes both edge and region information to segment object. It performs better than C-V method due to the edge constraints.

The edge-based level set methods achieve the best effect in the evaluation; in particular our hybrid method gets the highest accuracy and makes the least false segmentation (FPE = 0.12, FNE = 0.11, and SI = 0.88). The fast-marching method is time efficient but usually sacrifices accuracy and the boundary of object is not smooth and regular enough (Figure 6(c)). The geodesic active contour method is easy to cause oversegmentation where the boundary of pancreas is week or even is fractured (Figure 6(b)). 

The proposed hybrid level set method employs fast-marching method which is optimized by energy-decrement algorithm to generate optimal initial pancreas region, so that the initial contour can be located near the final one. The modified distance regular level set method maintains the desired shape of level set function in evolution, so that zero level set contour can regularly expand toward desired locations. An energy-tune algorithm is proposed to overcome problem of leakage in segmentation results. Moreover, a two-phase-segmentation scheme is employed in actual pancreas extraction process. The update of evolution function provides an effective way to control oversegmentation. Therefore, our hybrid method outperforms other evaluated methods in pancreas extraction by getting higher accuracy and less segmentation error.

On the time efficiency comparison, region growing method is faster than level set methods. Among all evaluated level set methods, fast-marching method is the fastest (0.197 ± 0.02 sec/slice); thus, it can quickly generate an optimal initial pancreas region for MDRLS. GAC method is the second fastest (0.465 ± 0.05 sec/slice). Since our method needs to deal with energy tune and maintains the distance regularization term, such that it takes 0.685 ± 0.05 sec/slice, which is slower than GAC method, C-V method and CER method need more calculation time due to the global information calculation.

## 5. Conclusion and Future Work

The proposed hybrid level set method effectively incorporates a fast-marching level set method and a modified distance regularized level set method to extract pancreas from CT image. Our main contribution is coming up with a feasible segmentation scheme and achieving better accuracy and time efficiency in pancreas extraction. Our hybrid level set method needs fewer and simple human-computer interaction. 

Based on energy-tune algorithm, the hybrid level set method overcomes the shortages of segmentation of object with nonideal edges in the complex texture of medical images. The modified distance regularized level set evolution provides stable and accurate numerical computation. Moreover, a two-phase-segmentation scheme is employed in MDRLS for further preventing the oversegmentation in pancreas region of nonideal edges. A fast-marching level set method employed in our method is able to generate optimal initial region for MDRLS in a short time while effectively improving segmentation speed. Therefore, the proposed hybrid level set method not only achieves accurate segmentation results but also is simultaneously time efficient.

In the future, we would apply the proposed hybrid level set method to extract other organs, such as liver, spleen, and heart. Moreover, we would utilize a priori knowledge including shape, location, and intensity distribution to guide the pancreas segmentation. A full-automatic algorithm is our next research target. 

## Figures and Tables

**Table 1:** Accurate evaluation values of FPE, FNE, and SI for each method.

Method	FPE	FNE	SI
FMDSL-SL	0.120256	0.112537	0.88384
FMESL-MSL	0.143215	0.135201	0.863634
CER	0.428885	0.227118	0.716948
C-V	0.503982	0.277937	0.669372
F-M	0.161511	0.33185	0.734745
GAC	0.321395	0.263718	0.764463
CCRG	1.26735	0.476753	0.468136
